# Kssdtree: an interactive Python package for phylogenetic analysis based on sketching technique

**DOI:** 10.1093/bioinformatics/btae566

**Published:** 2024-09-19

**Authors:** Hang Yang, Xiaoxin Lu, Jiaxing Chang, Qing Chang, Wen Zheng, Zehua Chen, Huiguang Yi

**Affiliations:** College of Computer Science and Technology (College of Data Science), Taiyuan University of Technology, Jinzhong 030600, China; Shenzhen Branch, Guangdong Laboratory for Lingnan Modern Agriculture, Genome Analysis Laboratory of the Ministry of Agriculture, Agricultural Genomics Institute at Shenzhen, Chinese Academy of Agricultural Sciences, Shenzhen 518055, China; Shenzhen Branch, Guangdong Laboratory for Lingnan Modern Agriculture, Genome Analysis Laboratory of the Ministry of Agriculture, Agricultural Genomics Institute at Shenzhen, Chinese Academy of Agricultural Sciences, Shenzhen 518055, China; College of Computer Science and Technology (College of Data Science), Taiyuan University of Technology, Jinzhong 030600, China; Shenzhen Branch, Guangdong Laboratory for Lingnan Modern Agriculture, Genome Analysis Laboratory of the Ministry of Agriculture, Agricultural Genomics Institute at Shenzhen, Chinese Academy of Agricultural Sciences, Shenzhen 518055, China; Shenzhen Branch, Guangdong Laboratory for Lingnan Modern Agriculture, Genome Analysis Laboratory of the Ministry of Agriculture, Agricultural Genomics Institute at Shenzhen, Chinese Academy of Agricultural Sciences, Shenzhen 518055, China; College of Computer Science and Technology (College of Data Science), Taiyuan University of Technology, Jinzhong 030600, China; College of Computer Science and Technology (College of Data Science), Taiyuan University of Technology, Jinzhong 030600, China; Shenzhen Branch, Guangdong Laboratory for Lingnan Modern Agriculture, Genome Analysis Laboratory of the Ministry of Agriculture, Agricultural Genomics Institute at Shenzhen, Chinese Academy of Agricultural Sciences, Shenzhen 518055, China

## Abstract

**Summary:**

Sketching technologies have recently emerged as a promising solution for real-time, large-scale phylogenetic analysis. However, existing sketching-based phylogenetic tools exhibit drawbacks, including platform restrictions, deficiencies in tree visualization, and inherent distance estimation bias. These limitations collectively impede the overall convenience and efficiency of the analysis. In this study, we introduce Kssdtree, an interactive Python package designed to address these challenges. Kssdtree surpasses other sketching-based tools by demonstrating superior performance in terms of both accuracy and time efficiency on comprehensive benchmarking datasets. Notably, Kssdtree offers key advantages such as intra-species phylogenomic analysis and GTDB-based phylogenetic placement analysis, significantly enhancing the scope and depth of phylogenetic investigations. Through extensive evaluations and comparisons, Kssdtree stands out as an efficient and versatile method for real-time, large-scale phylogenetic analysis.

**Availability and implementation:**

The Kssdtree Python package is freely accessible at https://pypi.org/project/kssdtree and source code is available at https://github.com/yhlink/kssdtree. The documentation and instantiation for the software is available at https://kssdtree.readthedocs.io/en/latest. The video tutorial is available at https://youtu.be/_6hg59Yn-Ws.

## 1 Introduction

Traditional phylogenomic analysis methods relied on sequence alignment tools such as BLAST (Altschul *et al.* 1990), CLUSTAL (Thompson *et al.* 1994), and MUSCLE (Edgar 2004). The advent of next-generation sequencing (NGS) techniques a decade ago significantly increased the volume and diversity of genomic data (Criscuolo and Gribaldo 2011), necessitating more efficient approaches. Alignment-free methods such as co-phylog (Yi and Jin 2013) and AAF (Fan *et al.* 2015) has emerged as essential alternatives, avoiding the time-consuming genome alignment process (Zielezinski *et al.* 2019). These methods typically process dozens of bacterial genomes in minutes—much faster than alignment-based methods, however, still struggle with large-scale bacterial genomes or medium-scale large eukaryotic genomes (Zielezinski *et al.* 2019).

Recently, sketching methods have emerged as promising solutions for large-scale comparative genomics and phylogenomic studies (Rowe 2019). These methods reduce genomic data to smaller *sketch* using specific *k*-mer sampling techniques, preserving the distance or similarity of genomes. For example, Mash (Ondov *et al.* 2016) can cluster 54 118 NCBI RefSeq genomes in 33 CPU hours, a feat beyond conventional alignment-free methods. Subsequent sketching methods, like SourMash (Pierce *et al.* 2019), BinDash (Zhao and Hancock 2019), and Kssd (Yi *et al.* 2021), have achieved even higher efficiency and accuracy.

It is essential to highlight that sketching methods alone can only provide distance estimations. To evolve into a phylogenomic analysis tool, sketching methods need to integrate downstream distance-based clustering algorithms, such as neighbor-joining (NJ) (Saitou and Nei 1987), dynamic neighbor-joining (DNJ) (Clausen and Schwartz 2023), and FastME (Lefort *et al.* 2015), along with tree visualization techniques such as ETE3 toolkit (Huerta-Cepas *et al.* 2016). Currently, Mashtree (Katz *et al.* 2019), which combines Mash with NJ, is the sole sketching-based phylogenetic analysis tool. However, Mashtree has notable limitations, including platform exclusivity, a lack of tree visualization, and distance estimation bias inherited from Mash (Ondov *et al.* 2016).

In this context, we introduce Kssdtree, a versatile Python package for phylogenetic analysis. Kssdtree offers superior accuracy and speed for large-scale phylogenomic analysis while enabling intra-species phylogenomic analysis and Genome Taxonomy Database (GTDB)-based phylogenetic placement analysis (Parks *et al.* 2022). In addition, it supports multiple operation system, runs seamlessly in a jupyter notebook environment, and provides interactive tree visualization.

## 2 Methods and materials

### 2.1 Genome sketching and distance estimation

Kssdtree is powered by the genome sketching technique Kssd (*k*-mer substring space sampling and decomposition), which pre-downsamples the whole *k*-mer space to a subset of *k*-mers that fit certain substring pattern (referred to as the *k*-mer substring subspace) and overlaps the *k*-mer substring subspace with a genome to create the genome sketch. In comparison, other genome sketching methods, such as Mash, Sourmash, and Bindash, sketch genomes by downsampling the *k*-mer hash spaces rather than the *k*-mer space (so-called minhash approaches). This can introduce extra bias for downstream distance estimation and set operations between sketches, since the mapping from *k*-mer spaces to the *k*-mer hash space is not exactly one-to-one (known as hash collision).

Suppose *S(A)* and *S(B)* are two sketches for genome *A* and *B*, respectively. The Jaccard index can be estimated by J(A, B)=|S(A)∩S(B)|/|S(A)∪S(B)|. And the Jaccard index can be converted to the evolutionary distance (*D*) using this formula proposed by Mash (Ondov *et al.* 2016):

(1) $$D=-\frac{1}{k}ln\frac{2J(A,\;B)}{1+J(A,\;B)},$$

where *k* is the length of *k*-mer.

### 2.2 Kssdtree pipelines

**Figure 1.**
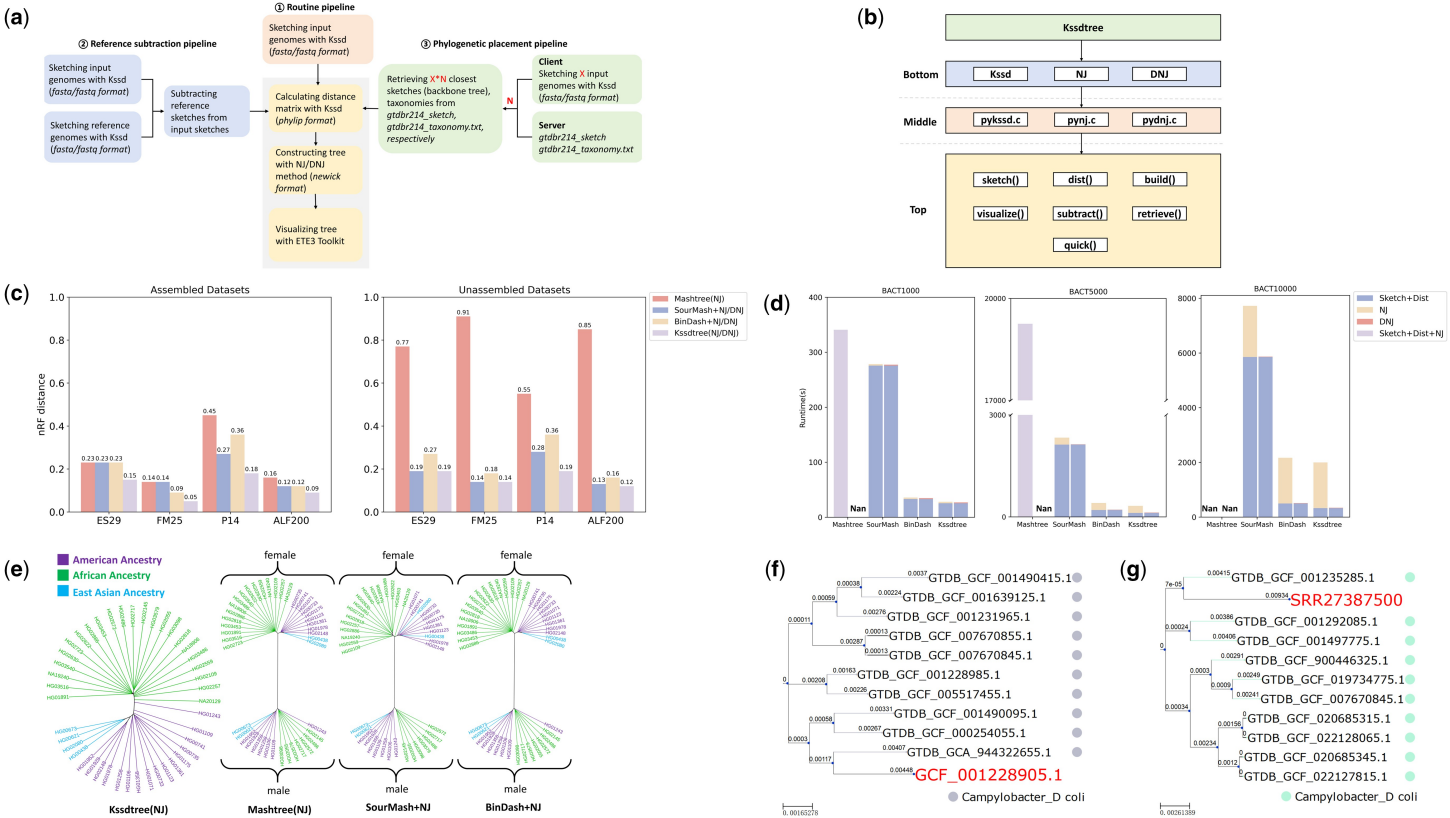
Overview of the Kssdtree pipelines, implementation, and performance. **(a)** Kssdtree pipelines: Routine pipeline, Reference subtraction pipeline, and GTDB-based phylogenetic placement pipeline. **(b)** Kssdtree implementation. **(c)** The normalized Robinson-Foulds (nRF) distances between truth and trees inferred by different methods. **(d)** Time efficiencies of different methods. The Sketch, Dist, and NJ steps of Mashtree cannot be separately measured, hence, the total time is shown. Mashtree does not support DNJ algorithm and reported failure on the BACT10000 dataset, as indicated by the presence of Nan (not a number) values. **(e)** The 43 human genomes phylogenomic trees constructed by different methods. Kssdtree phylogenetic placement analysis of **(f)** an assembled genome *GCF_001228905.1* and **(g)** a raw sequencing run *SRR27387500* from species *Campylobacter coli.*

Kssdtree provides three distinct pipelines tailored for specific types of phylogenetic analyses (see Fig. 1a): Routine pipeline, Reference subtraction pipeline, and GTDB-based phylogenetic placement pipeline.

Routine pipeline provides a general-purpose phylogenomic analysis that consists of these steps (see Supplementary Fig. S1 for details):

1. Sketch genomes with Kssd using the predefined *k*-mer substring subspace (*.shuf file).
2. Calculate distances with Kssd [see Equation (1)] and create distance matrix in *phylip* format.
3. Generate *newick* format tree file using the NJ or DNJ method based on the distance matrix.
4. Visualize the tree using ETE3 toolkit.

The reference subtraction pipeline is designed for intra-species genome clustering analysis. The steps involved are:

1. Select and sketch a representative reference genome of the species.
2. Sketch individual genomes that from the same species.
3. Subtract the reference sketch from each individual genome sketch.
4. Conduct distance estimation, tree generation, and visualization in the same manner as the routine pipeline, but using the remainder sketches generated in (3).

The GTDB-based phylogenetic placement pipeline allows retrieving the most similar genomes from GTDB and performing phylogenomic analysis using the retrieved genomes together with the query genomes. To achieve this, all 402 709 genomes of GTDB (Release 08-RS214) were pre-sketched using Kssd, resulting in a sketch database *gtdbr214_sketch* on our remote server. When user provides their query genomes, the pipeline sketches all the query genomes and send the sketch to the server, and *N* most similar genomes from the sketch database are retrieved for each query genome based on its distance to each genome in the database [see Equation (1)]. Then all the query genomes together their most similar genomes subject to phylogenomic analysis in the same way as in the routine pipeline. The phylogenetic placement of the query genome on the prokaryotic tree of life can be approximately identified by searching their most similar genomes on the GTDB tree database (https://gtdb.ecogenomic.org/tree).

### 2.3 Implementation

The Kssdtree modules are implemented in a three-level hierarchy, optimizing efficiency and user-friendliness (see Fig. 1b).

1. Bottom Level: Consists of the existing core functions of Kssd, NJ, and DNJ algorithms, which were written in C.
2. Middle Level: Core functions are encapsulated in Cython (Behnel *et al.* 2011) modules *pykssd.c*, *pynj.c*, and *pydnj.c* for seamless interaction between the top and bottom levels.
3. Top Level: A user-friendly Python interface featuring a diverse array of functions for phylogenetic analysis, including *sketch*, *dist*, *build*, and *visualize*. It also includes functions specific to the reference subtraction and GTDB-based phylogenetic placement pipelines, such as *subtract* and *retrieve*. In addition, the core *quick* function is designed to simplify the three pipelines, eliminating the need for users to manually organize numerous intermediate files.

### 2.4 Benchmarking

We compared both accuracy and efficiency of Kssdtree (v2.0.2) with three other sketching-based methods of the latest release: Mashtree (v1.2.0), SourMash (v4.8.2) + NJ/DNJ, and BinDash (v0.2.1) + NJ/DNJ. In the three methods, only Mashtree can generated phylogenetic tree file in *newick* format, whereas SourMash and BinDash can only output the pairwise distances. Therefore, we need to manually pipe SourMash and BinDash outputs to NJ or DNJ algorithms to construct phylogenetic tree. The two pipelines are named as SourMash+NJ/DNJ and BinDash+NJ/DNJ, respectively, in this study.

We collected both real and simulated benchmarking datasets. The real datasets including 29 *Escherichia coli/Shigella* genomes (ES29), 25 fish mitochondrial genomes (FM25), and 14 plant species (P14), which were specifically designed for phylogenetic tool benchmarking, were download from the AF project (Zielezinski *et al.* 2019) (https://afproject.org/app/). The simulated dataset consisted of 200 species produced by the ALF simulator (Dalquen *et al.* 2012) (ALF200, see Supplementary Section S1.1 for details). In addition, the unassembled raw reads of the four datasets were generated using the DWGSIM sequencing data simulator (https://github.com/nh13/DWGSIM) (see Supplementary Section S1.2 for details).

To benchmark the running time efficiency, 1000, 5000, and 10 000 bacterial genomes from NCBI RefSeq were randomly sampled from the latest NCBI bacteria Refseq (https://www.ncbi.nlm.nih.gov/refseq/), denoted as BACT1000, BACT5000, and BACT10000, respectively. For the benchmarking of intra-species phylogenomic analysis, 43 relatively completed human genomes (HG43) were download from the recently published human pangenome reference (Liao *et al.* 2023) (https://s3-us-west-2.amazonaws.com/human-pangenomics/index.html) (see Supplementary Section S1.3 for the metadata). All the methods were tested on a 32GB, 12-core machine.

## 3 Results

### 3.1 Accuracy

We measured the accuracy of an inferred tree using its normalized Robinson-Foulds (nRF) distance to the ground-truth tree. The nRF (Robinson and Foulds 1981) is a metric ranging from 0 to 1, where 0 indicates identical tree topologies, while 1 indicates the most dissimilar topologies. The nRF distances of all methods were assessed using the *compare* function in the ETE3 toolkit while controlling for *k*-mer length (*k*) and sketch size (*s*) (see Supplementary Section S1.4 for details). The findings, illustrated in Fig. 1c (refer to Supplementary Section S1.5 for the ground-truth trees and the inferred trees), showed that Kssdtree consistently demonstrated the smallest nRF distances across all benchmarking datasets, encompassing both real and simulated scenarios as well as assembled and unassembled datasets. These results underscored the suitability of Kssdtree for diverse datasets, affirming its superiority among sketching-based tools and its applicability to both assembled and unassembled genomic data. For details on accessing these tools, refer to the experiment scripts in Supplementary Section S1.6.1.

### 3.2 Time efficiency

Speed testing based on three benchmarking datasets (BACT1000, BACT5000, and BACT10000) revealed that Kssdtree exhibits slightly higher time efficiency than BinDash+NJ/DNJ, while both Kssdtree and BinDash+NJ/DNJ significantly outperform other sketching-based methods, as illustrated in Fig. 1d. These findings also underscore the importance of using DNJ when working with large datasets: Mashtree, a method equipped with a built-in NJ algorithm, encountered difficulties in completing the tree construction process for the BACT10000 dataset. For methods other than Mashtree, the utilization of the optimal DNJ algorithm significantly improved the speed compared to using NJ algorithm when the scales of datasets are very large (e.g. BACT5000 and BACT10000). All tools compared here adhered to the same *k*-mer length and dimensionality-reduction rates, ensuring a fair and consistent comparison (see Supplementary Section S1.4 for details). The experiment scripts for evaluating these tools can be found in Supplementary Section S1.6.2.

### 3.3 Intra-species phylogenomic analysis

Genomes from the same species are highly similar and possess relatively sparse differential *k*-mers that are phylogenetically informative. After genome sketching, the number of differential *k*-mers decreases further, which can affect the accuracy of phylogenomic analysis. As a solution, Kssdtree augments the representation of differential *k*-mers in genomes though the reference subtraction pipeline (refer to Methods).

We compared the performance of Kssdtree against other sketching-based methods using 43 human genomes from diverse populations (the HG43 dataset). The results showed that other sketching-based methods tended to cluster genomes based on gender. This tendency arises because the differences among human genomes mainly stem from the sex chromosomes, and these methods do not support reference subtraction operations. Conversely, Kssdtree, capable of sketch subtraction operations, subtracts the reference sketch (including sex chromosomes) from individual genome sketches before distance computation and tree construction. This approach allows Kssdtree to organize genomes unambiguously by population, irrespective of gender influence.

When controlling for gender, Kssdtree still demonstrated superior performance, unequivocally grouping genomes based on their population origins. In contrast, other methods exhibited limitations. For example, Mashtree, BinDash+NJ, and SourMash+NJ trees failed to cluster male genomes of the American population and female genomes of the East Asian population into cohesive groups (see Fig. 1e and Supplementary Section S1.6.3 for details).

### 3.4 GTDB-based phylogenetic placement

We tested the GTDB-based phylogenetic placement pipeline of Kssdtree using both complete genome and unassembled raw reads from the randomly selected species *Campylobacter coli*. Kssdtree correctly identified the originating species for the query genome based on the taxonomy of the most similar genomes retrieved. The phylogenetic relationships revealed between the query genome and its most similar genomes from GTDB (Fig. 1f and g) allowed us to determine the phylogenetic placement of the query genome within the prokaryotic tree of life using the GTDB tree database. Finally, we summarized the feature enhancements brought by Kssdtree in Table 1.

**Table 1.** Supported features of different methods.

Support		Mashtree	SourMash+NJ	BinDash+NJ	Kssdtree
Platform	Linux	YES	YES	YES	YES
	MacOS	YES	YES	YES	YES
	Windows	NO	YES	YES	YES
One-stop tree construction		YES	NO	NO	YES
Tree visualization		NO	NO	NO	YES
Intra-species phylogenomic analysis		NO	NO	NO	YES
GTDB-based phylogenetic placement		NO	NO	NO	YES

## 4 Conclusions

In this paper, we present Kssdtree, a versatile Python package for phylogenetic analysis that can be operated on a personal computer. Kssdtree supports gzip-compressed and uncompressed *fasta* and *fastq* files. Compared to other sketching-based methods, Kssdtree shows superior performance in terms of accuracy on both assembled and unassembled genomic data. Notably, Kssdtree offers optimized pipelines for intra-species phylogenomic analysis and GTDB-based phylogenetic placement.

## Supplementary Material

btae566_Supplementary_Data

## Data Availability

The data underlying this article are available in AF project at https://afproject.org/app, NCBI bacteria Refseq at https://www.ncbi.nlm.nih.gov/refseq, and human genomes at https://zenodo.org/uploads/12699159.
