# Supplementary material for: Kssdtree: an interactive Python package for phylogenetic analysis based on sketching technique
Source: Bioinformatics. 2024 Sep 19;40(10):btae566. doi: 10.1093/bioinformatics/btae566 (PMC11467128; doi:10.1093/bioinformatics/btae566)
Supplement: btae566_Supplementary_Data [file btae566_supplementary_data.pdf]

Supplementary Materials

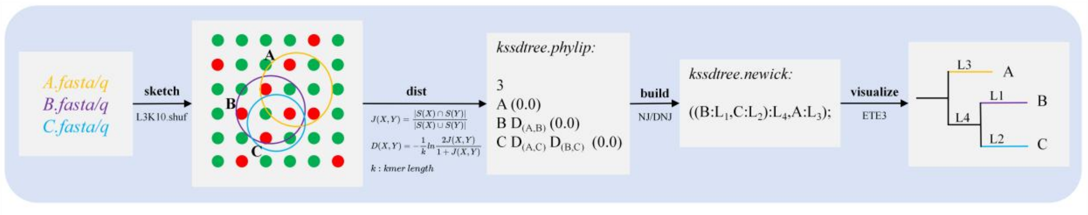

Figure S1: Overview the routine pipeline of Kssdtree. Step 1) Sketch genomes with Kssd. These genomes were then sketched using a half k-mer length of 10 and a 4096-fold dimensionality reduction rate ("L3K10.shuf"); Step 2) Calculate distances with Kssd and create distance matrix in phylip format. A subset of k-mers s termed k-mer subspace (shown as red dots here) are drawn randomly from k-mer space S (namely the collection of all possible string of length k defined in a given alphabet set, shown as green dots and red dots here). then, the sketch of any given sequence is built by overlapping s with the k-mers set of this sequence. After sketching, This allows for the straightforward estimation of pairwise similarity using the Jaccard index and then further convert the Jaccard index to evolutionary distance. After the evolutionary distance of all input genomes is calculated, a phylip format distance matrix is created; Step 3) Construct tree with the NJ or DNJ method using the distance matrix and generate tree in newick format; Step 4) Visualize the tree with ETE3 toolkit.

1. Experiment Sections

1.1 ALF200 simulation

The parameters for ALF simulation were set in *alf-params.drw*, and the other parameters follow the default setting.

```
alf-params.drw:
### parameters for root genome
# number of proteins first organism have
protStart := 50;
# parameters for the gene length distribution (~Gamma(k, theta))
gammaLengthDist := [2.4, 133.8];
# minimum length of a gene
minGeneLength := 20000;
###

### substitution models
substModels := [
SubstitutionModel('CPAM'),
SubstitutionModel('TN93', [.3, .4, .7], [seq(0.25,4)], true),
NULL
]:
# when no substitution model is given (pure gap simulation), select block size for gaps
blocksize := 3:
```

```
###

### tree parameters
# BDTree, ToLSample, Custom
treeType := 'BDTree':
# PAM distance from origin to recent species (for random trees)
mutRate := 100;
# scale tree to match Pam distance defined below (parameter mutRate)
scaleTree := false:
# b parameter (for BDTree)
birthRate := 0.01:
# d parameter (for BDTree)
deathRate := 0.001:
# number of species in the tree (for BD and ToL)
NSpecies := 200:
# for BDTree: should resulting tree be ultrametric
ultrametric := false:
...
```

Run it with the following command: bin/alfsim alf-params.drw

1.2 Raw sequencing data simulation

The unassembled raw reads of the four datasets (ES29, FM25, P14, and ALF200) were generated using the DWGSIM with *simulate_sequencing.sh* script.

```
simulate_sequencing.sh:
# Folder: the path of four assembled datasets (ES29, FM25, P14 and ALF200)
DIR="Folder"
# sequencing coverage
COVERAGE=2
for fasta_file in "$DIR"/*.fasta
do
    # Extract the file name from the path
    filename=$(basename "$fasta_file")
    # Remove the .fasta extension from file name
    file_basename="${filename%.fasta}"
    # the sequence length
    seq_length=$(grep -v ">" "$DIR/$filename" | tr -d "\n" | wc -c)
    N=$((COVERAGE * seq_length/150))
    # Run dwgsim to simulate sequencing
    dwgsim -N $N -1 150 -2 150 -y 0 "$DIR/$filename" "$DIR/$file_basename"
    echo "sequence len: $seq_length"
    echo "reads nums: $N"
    echo "File name: $filename"
    echo "File base name: $file_basename"
done
```

Run it with the following command: ./simulate_sequencing.sh

1.3 HG43 dataset

Table S1. The metadata of 43 human genomes

Sample	Cohort	Sex	Superpopulation ID	Superpopulation Name
HG01123	HPRC	female	AMR	American Ancestry
HG01258	HPRC	male	AMR	American Ancestry
HG01358	HPRC	male	AMR	American Ancestry
HG01361	HPRC	female	AMR	American Ancestry
HG00735	HPRC	female	AMR	American Ancestry
HG00741	HPRC	female	AMR	American Ancestry
HG01071	HPRC	female	AMR	American Ancestry
HG01106	HPRC	male	AMR	American Ancestry
HG01175	HPRC	female	AMR	American Ancestry
HG00733	HPRC_PLUS	female	AMR	American Ancestry
HG01109	HPRC_PLUS	male	AMR	American Ancestry
HG01243	HPRC_PLUS	male	AMR	American Ancestry
HG01928	HPRC	male	AMR	American Ancestry
HG01952	HPRC	male	AMR	American Ancestry
HG01978	HPRC	female	AMR	American Ancestry
HG02148	HPRC	female	AMR	American Ancestry
HG01891	HPRC	female	AFR	African Ancestry
HG02257	HPRC	female	AFR	African Ancestry
HG02486	HPRC	male	AFR	African Ancestry
HG02559	HPRC	female	AFR	African Ancestry
HG02109	HPRC_PLUS	female	AFR	African Ancestry
HG02145	HPRC_PLUS	male	AFR	African Ancestry
HG02055	HPRC_PLUS	male	AFR	African Ancestry
HG02572	HPRC	male	AFR	African Ancestry
HG02622	HPRC	female	AFR	African Ancestry
HG02630	HPRC	female	AFR	African Ancestry
HG02717	HPRC	male	AFR	African Ancestry
HG02886	HPRC	female	AFR	African Ancestry
HG03540	HPRC	female	AFR	African Ancestry
HG02723	HPRC_PLUS	female	AFR	African Ancestry
HG02818	HPRC_PLUS	female	AFR	African Ancestry
HG03453	HPRC	female	AFR	African Ancestry
HG03579	HPRC	male	AFR	African Ancestry

HG03486	HPRC_PLUS	female	AFR	African Ancestry
HG03098	HPRC_PLUS	male	AFR	African Ancestry
NA18906	HPRC_PLUS	female	AFR	African Ancestry
NA19240	HPRC_PLUS	female	AFR	African Ancestry
NA20129	HPRC_PLUS	female	AFR	African Ancestry
HG03516	HPRC	female	AFR	African Ancestry
HG00438	HPRC	female	EAS	East Asian Ancestry
HG00621	HPRC	male	EAS	East Asian Ancestry
HG00673	HPRC	male	EAS	East Asian Ancestry
HG02080	HPRC_PLUS	female	EAS	East Asian Ancestry

1.4 Experiment parameters

Table S2. The experiment parameters of different methods under different datasets

Dataset		Mashtree(NJ)	SourMash+NJ/DNJ	BinDash+NJ/DNJ	Kssdtree(NJ/DNJ)
Assembled	ES29	k=20, s=1185	k=20, scaled=4096	k=20, s=1216 (19)	L3K10.shuf
	FM25	k=12, s=1009	k=12, scaled=16	k=12, s=1024 (16)	L1K6.shuf
	P14	k=22, s=54342	k=22, scaled=4096	k=22, s=849	L3K11.shuf
	ALF200	k=16, s=8603	k=16, scaled=256	k=16, s=134	L2K8.shuf
	BACT1000	k=18, s=12285	k=18, scaled=4096	k=18, s=192	L3K9.shuf
	BACT5000	k=18, s=15117	k=18, scaled=4096	k=18, s=236	L3K9.shuf
	BACT10000	k=18, s=15796	k=18, scaled=4096	k=18, s=247	L3K9.shuf
	HG43	k=20, s=530550	k=20, scaled=4096	k=20, s=8289	L3K10.shuf
Unassembled	ES29	k=20, s=2451	k=20, scaled=4096	k=20, s=38	L3K10.shuf
	FM25	k=12, s=1747	k=12, scaled=16	k=12, s=27	L1K6.shuf
	P14	k=22, s=115063	k=22, scaled=4096	k=22, s=1798	L3K11.shuf
	ALF200	k=16, s=18168	k=16, scaled=256	k=16, s=279	L2K8.shuf

Note: The k and s represent the k-mer length and sketch size, respectively. Both Mashtree and BinDash use the average sketch size after Kssdtree dimension reduction, where the sketch size of BinDash is the nearest multiple of 64. The scaled factor of SourMash is consistent with the dimensionality reduction rate of Kssdtree (scaled=16: L1K*.shuf, scaled=256: L2K*.shuf, scaled=4096: L3K*.shuf).

1.5 Ground-truth trees and inferred trees

Figure S2-S9: The benchmark tree and the trees constructed by the four sketching-based methods, Kssdtree(NJ/DNJ), Mashtree(NJ), SourMash+NJ/DNJ and BinDash+NJ/DNJ, on the assembled and unassembled datasets (ES29, FM25, P14, and ALF200).

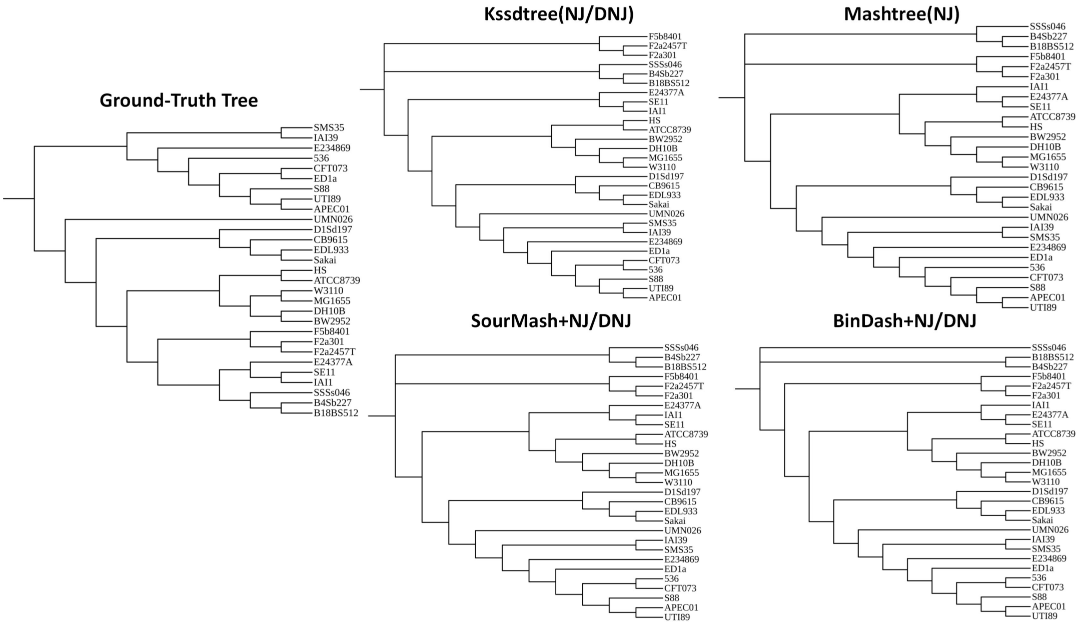

Figure S2. The ground-truth tree and the trees constructed by the four methods on the assembled ES29 dataset.

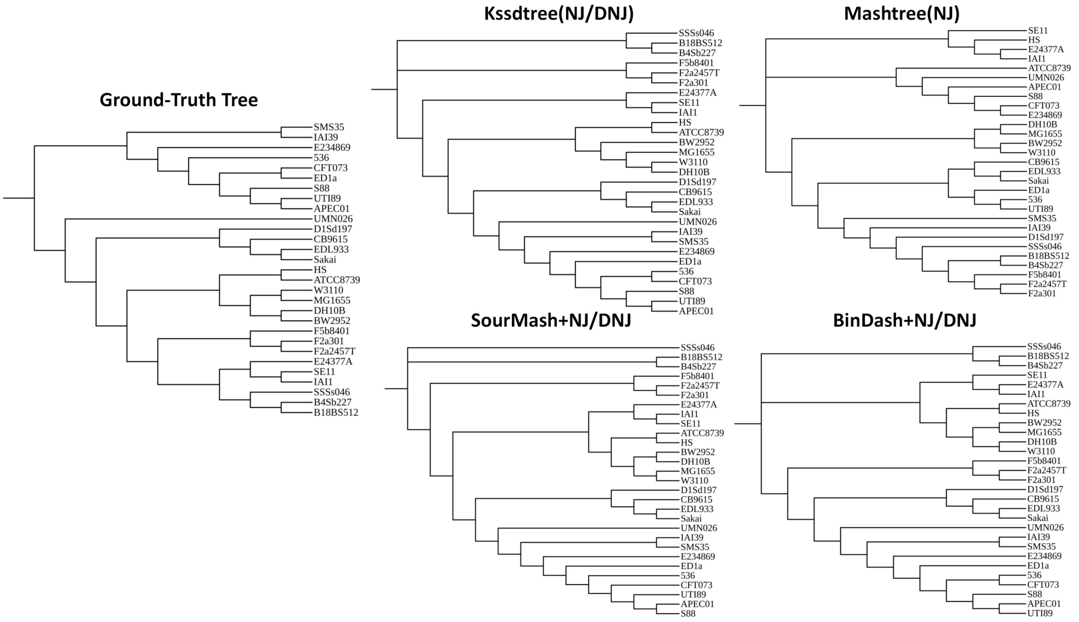

Figure S3. The ground-truth tree and the trees constructed by the four methods on the unassembled ES29 dataset.

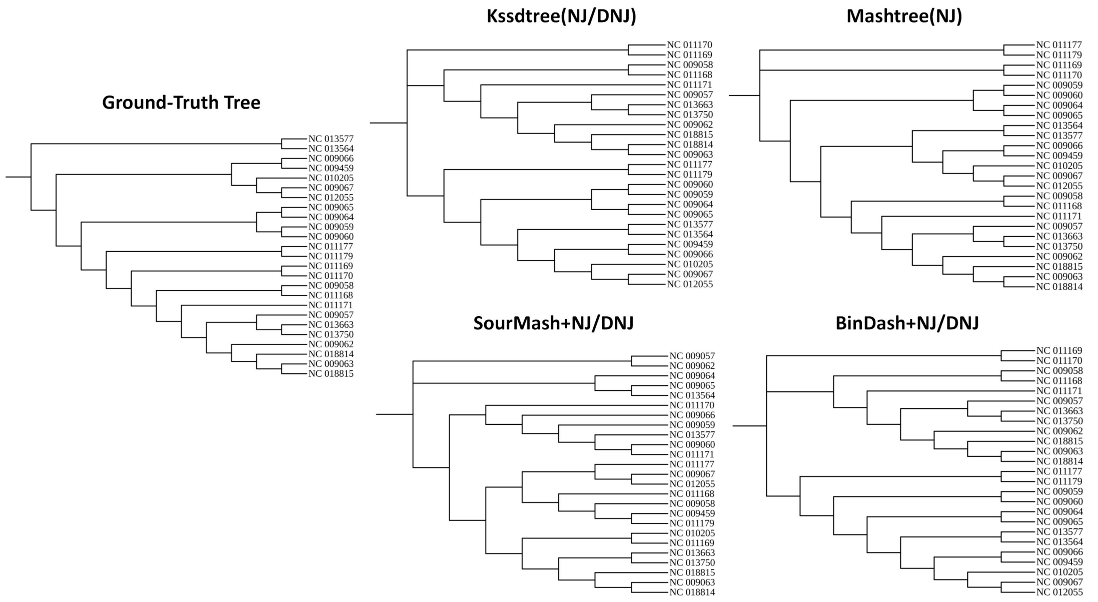

Figure S4. The ground-truth tree and the trees constructed by the four methods on the assembled FM25 dataset.

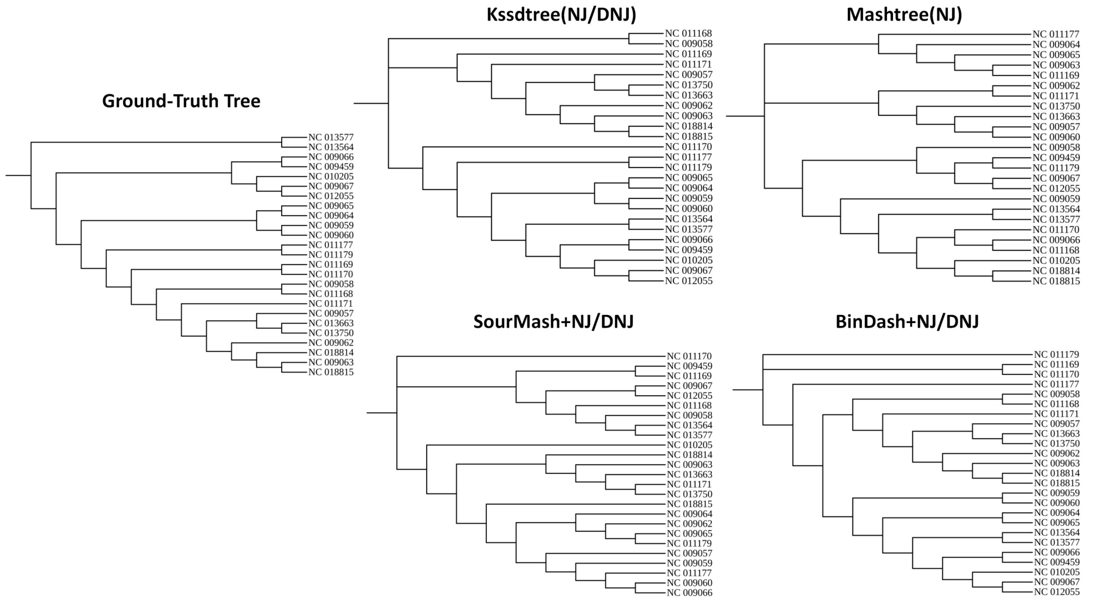

Figure S5. The ground-truth tree and the trees constructed by the four methods on the unassembled FM25 dataset.

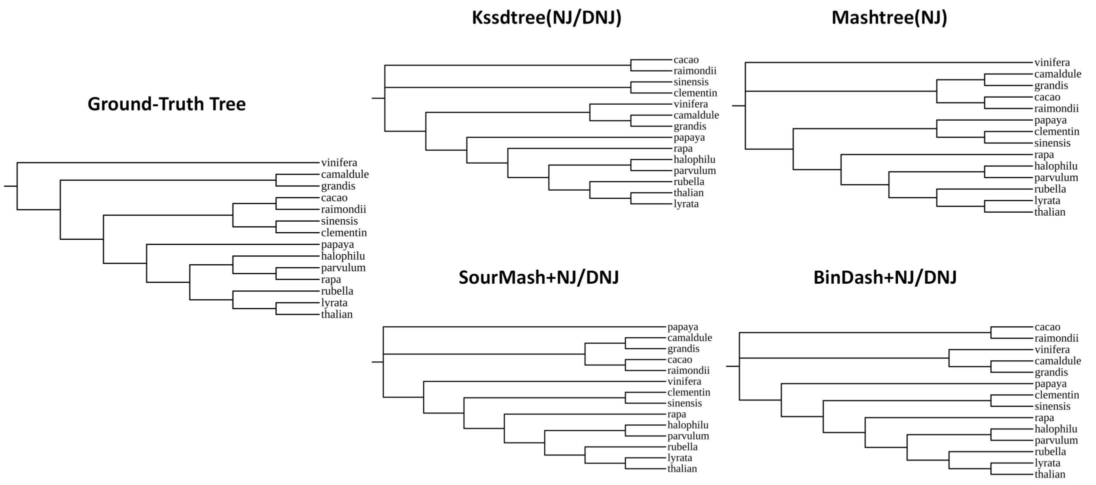

Figure S6. The ground-truth tree and the trees constructed by the four methods on the assembled P14 dataset.

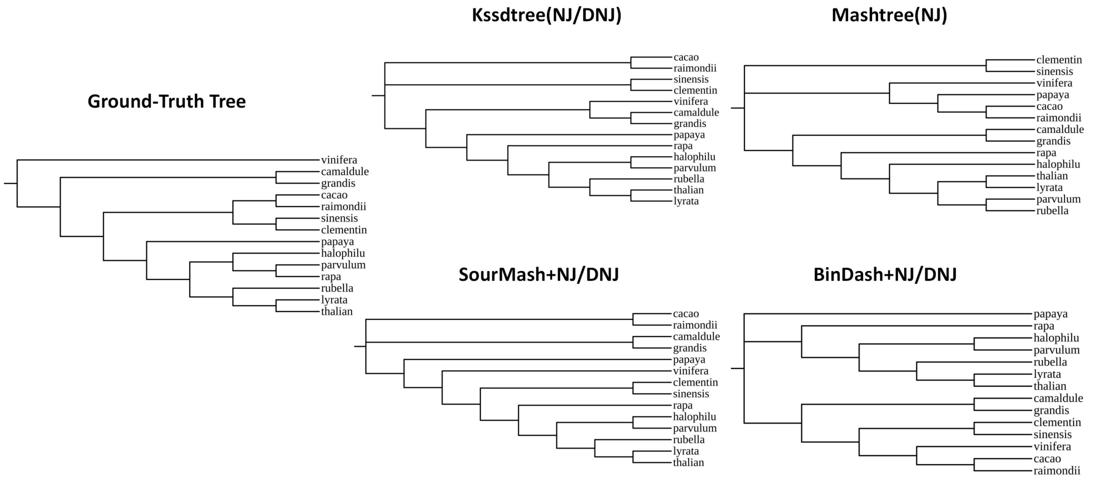

Figure S7. The ground-truth tree and the trees constructed by the four methods on the unassembled P14 dataset.

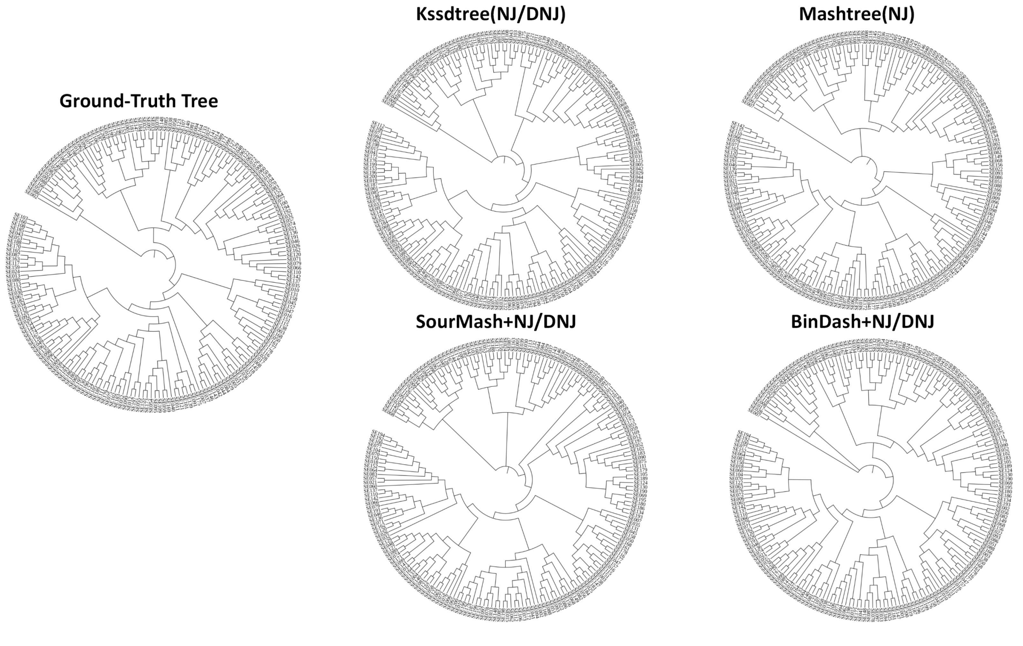

Figure S8. The ground-truth tree and the trees constructed by the four methods on the assembled ALF200 dataset.

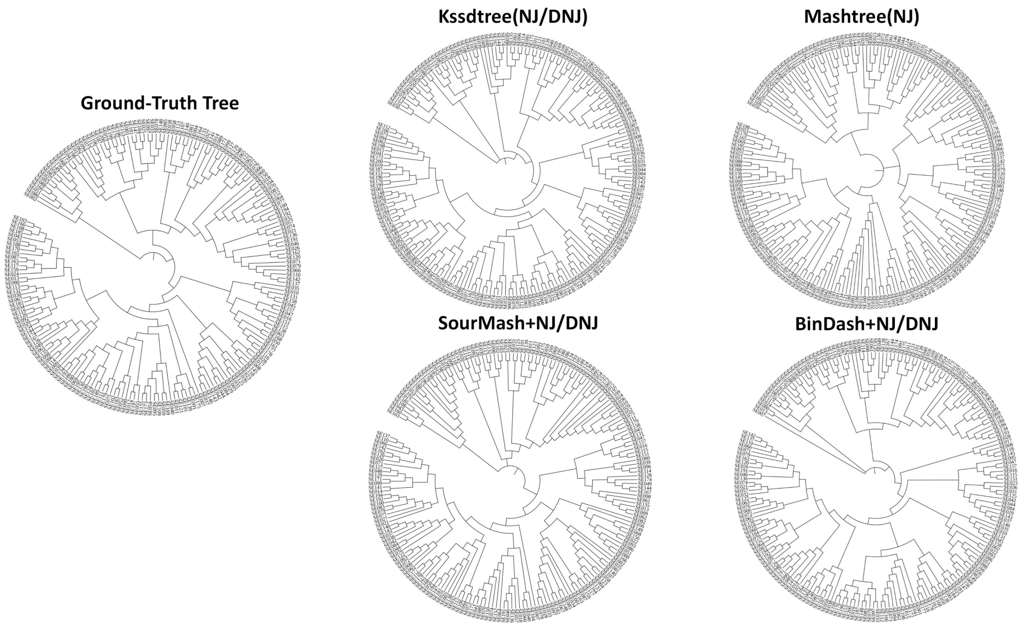

Figure S9. The ground-truth tree and the trees constructed by the four methods on the unassembled ALF200 dataset.

1.6 Tool comparison scripts

1.6.1 Accuracy

Below are the commands that can be used to compare sketching-based methods on the assembled ES29 dataset. The parameters in the other assembled and unassembled datasets (ES29, FM25, P14, and ALF200) are shown in Table S2.

```
# Kssdtree (NJ/DNJ)
kssdtree.quick(shuf_file='L3K10.shuf', genome_files='ES29', output='kssdtree.newick', method='nj')
kssdtree.quick(shuf_file='L3K10.shuf', genome_files='ES29', output='kssdtree.newick', method='dnj')

# Mashtree (NJ)
mashtree --kmerlength 20 --sketch-size 1185 --mindepth 0    ES29/* > mashtree.newick

# SourMash+NJ/DNJ
sourmash sketch dna -p k=20,scaled=4096 -o ES29.sig ES29/*
sourmash compare ES29.sig --csv ES29_distance.csv
python create_matrix_sourmash.py
kssdtree.build(phylip= 'ES29_matrix.phy', output= 'sourmashtree.newick', method='nj')
kssdtree.build(phylip= 'ES29_matrix.phy', output= 'sourmashtree.newick', method='dnj')

# BinDash+NJ/DNJ
bindash sketch --kmerlen=20 --sketchsize64=19 --outfname=ES29.sketch ES29/*
bindash dist --mthres=1 --outfname=ES29_distance.out ES29.sketch
python create_matrix_bindash.py
```

```
kssdtree.build(phylip= 'ES29_matrix.phy', output= 'bindashtree.newick', method='nj')
kssdtree.build(phylip= 'ES29_matrix.phy', output= 'bindashtree.newick', method='dnj')

# Compute nRF distance
ete3 compare -t kssdtree.newick mashtree.newick sourmashtree.newick bindashtree.newick -r RealTree.newick
--unrooted
```

1.6.2 Time efficiency

Below are the commands that can be used to compare sketching-based methods on the BACT1000 dataset. The parameters in the other datasets (BACT5000 and BACT10000) are shown in Table S2.

```
# Kssdtree (NJ)
# Sketch: Sketch 1000 bacteria genomes (BACT1000)
py_sketch.py:
import kssdtree
kssdtree.sketch(shuf_file='L3K9.shuf', genome_files='BACT1000', output='BACT1000_sketch')

/usr/bin/time -v python py_sketch.py

# Dist: Compute pairwise distance and generate distance matrix
py_dist.py:
import kssdtree
kssdtree.dist(genome_sketch='BACT1000_sketch', output='BACT1000.phy', flag=0)

/usr/bin/time -v python py_dist.py

# NJ: Generate tree file with NJ method
py_bulid.py:
import kssdtree
kssdtree.build(phylip='BACT1000.phy', output='BACT1000.newick', method='nj')

/usr/bin/time -v python py_bulid.py

# Kssdtree (DNJ)
# Sketch: Sketch 1000 bacteria genomes (BACT1000)
py_sketch.py:
import kssdtree
kssdtree.sketch(shuf_file='L3K9.shuf', genome_files='BACT1000', output='BACT1000_sketch')

/usr/bin/time -v python py_sketch

# Dist: Compute pairwise distance and generate distance matrix
py_dist.py:
import kssdtree
kssdtree.dist(genome_sketch='BACT1000_sketch', output='BACT1000.phy', flag=1)
```

```
/usr/bin/time -v python py_dist.py

# DNJ: Generate tree file with DNJ method
py_bulid.py:
import kssdtree
kssdtree.build(phylip='BACT1000.phy', output='BACT1000.newick', method='dnj')

/usr/bin/time -v python py_bulid.py

# Mashtree (NJ)
# Sketch+Dist+NJ: Sketch 1000 bacteria genomes, compute pairwise distance and generate distance matrix,
and generate tree file with NJ method
/usr/bin/time -v mashtree --kmerlength 18 --sketch-size 12285 --mindepth 0 --numcpus 12 BACT1000/* >
BACT1000.newcick

# SourMash+NJ/DNJ
# Sketch: Sketch 1000 bacteria genomes (BACT1000)
/usr/bin/time -v sourmash sketch dna -p k=18,scaled=4096 -o BACT1000.sig BACT1000/*

# Dist: Compute pairwise distance and generate distance matrix
/usr/bin/time -v sourmash compare BACT1000.sig -p 12 --csv BACT1000_distance.csv
/usr/bin/time -v python create_matrix_sourmash.py

# NJ/DNJ: Generate tree file with the NJ/DNJ method taking the same time as Ksstree
...

# BinDash+NJ/DNJ
# Sketch: Sketch 1000 bacteria genomes (BACT1000)
/usr/bin/time -v bindash sketch --kmerlen=18 --sketchsize64=192 --nthreads=12
--outfname=BACT1000.sketch BACT1000/*

# Dist: Compute pairwise distance and generate distance matrix
/usr/bin/time -v bindash dist --nthreads=12 --mthres=1 --outfname=BACT1000_distance.out
BACT1000.sketch
/usr/bin/time -v python create_matrix_bindash.py

# NJ/DNJ: Generate tree file with the NJ/DNJ method taking the same time as Ksstree
...
```

1.6.3 Population level phylogenomics

Below are the commands that can be used to compare sketching-based methods on the HG43 dataset.

```
# Kssdtree (NJ)
# Sketch human reference genome (hg38.fa.gz)
```

```
ksstree.sketch(shuf_file='L3K10.shuf', genome_files='hg38.fa.gz', output='Ref_sketch', set_opt=True)

# Sketch 43 human genomes (HG43)
ksstree.sketch(shuf_file='L3K10.shuf', genome_files='HG43', output='HG43_sketch', set_opt=True)

# Subtract Ref_sketch from HG43_sketch
ksstree.subtract(ref_sketch='Ref_sketch', genome_sketch='HG43_sketch', output='HG43_sub_sketch')

# Compute pairwise distance and generate distance matrix
ksstree.dist(genome_sketch='HG43_sub_sketch', output='HG43_matrix.phy', flag=0)

# Generate tree file with NJ method
ksstree.build(phylip='HG43_matrix.phy', output='HG43.newick', method='nj')

# The above process is equivalent to the following simplified version
kssdtree.quick(shuf_file='L3K10.shuf', genome_files='HG43', output='HG43.newick',
reference='hg38.fa.gz', method='nj')

# Mashtree (NJ)
# Sketch 43 human genomes (HG43), compute pairwise distance and generate distance matrix, and generate
tree file with NJ method
mashtree --kmerlength 20 --sketch-size 530550 --mindepth 0 --numcpus 12 HG43/* > HG43.newick

# SourMash+NJ
# Sketch 43 human genomes (HG43)
sourmash sketch dna -p k=20,scaled=4096 -o HG43.sig HG43/*

# Compute distance and generate distance matrix
sourmash compare HG43.sig -p 12 --csv HG43_distance.csv
python create_matrix_sourmash.py

# Generate tree file with NJ method
kssdtree.build(phylip= 'HG43_matrix.phy', output= 'HG43.newick', method='nj')

# BinDash+NJ
# Sketch 43 human genomes (HG43)
bindash sketch --kmerlen=20 --sketchsize64=8289 --nthreads=12 --outfname=HG43.sketch HG43/*

# Compute pairwise distance and generate distance matrix
bindash dist --nthreads=12 --mthres=1 --outfname=HG43_distance.out HG43.sketch
python create_matrix_bindash.py

# Generate tree file with NJ method
kssdtree.build(phylip= 'HG43_matrix.phy', output= 'HG43.newick', method='nj')
```

Note: The above *create_matrix_sourmash.py* and *create_matrix_bindash.py* scripts are used to create distance matrix for SourMash and BinDash, respectively.

```
create_matrix_sourmash.py:
import pandas as pd
import math
def deal_sourmash(k, sourmash_distance_path, output):
    print('deal sourmash...')
    with open(sourmash_distance_path) as f:
        first_line = f.readline().strip()
    temp_names = first_line.split(',')
    seq_names = []
    for x in temp_names:
        if '.fasta.gz' in x or '.fastq.gz' in x:
            seq_names.append(x.split('/')[-1][:-9])
        elif '.fna.gz' in x:
            seq_names.append(x.split('/')[-1][:-7])
        elif '.fa.gz' in x:
            seq_names.append(x.split('/')[-1][:-6])
        elif x[-3:] == '.fa':
            seq_names.append(x.split('/')[1][:-3])
        elif x[-6:] == '.fasta' or x[-6:] == '.fastq':
            seq_names.append(x.split('/')[1][:-6])
        else:
            pass
    data = pd.read_csv(sourmash_distance_path, header=None, skiprows=[0])
    with open(output, 'w') as f:
        f.write(str(len(seq_names)) + '\n')
        for i in range(len(seq_names)):
            f.write(seq_names[i])
            for j in range(len(seq_names)):
                if i == j:
                    f.write('\t{:.5f}'.format(0.0))
                else:
                    jaccard = data[i][j]
                    if jaccard == 0:
                        mashD = 1
                    else:
                        mashD = -(1 / k) * math.log(2 * jaccard / (1 + jaccard))
                    f.write('\t{:.5f}'.format(mashD))
            f.write('\n')

if __name__ == '__main__':
    deal_sourmash(20, 'ES29_distance.csv', 'ES29_matrix.phy')
```

```
create_matrix_bindash.py:
import pandas as pd
import os
import math
def deal_bindash(k, bindash_distance_path):
    print('deal bindash...')
    df = pd.read_csv(bindash_distance_path, delimiter='\t', header=None)
    df.columns = ['Ref', 'Qry', 'MashD', 'P-value', 'Matching-hashes']
    new_df = df[['Qry', 'Ref', 'Matching-hashes']]
    new_df['Matching-hashes'] = new_df['Matching-hashes'].apply(lambda x: int(x.split('/')[0]) / int(x.split('/')[1]))
    if '.fasta.gz' in new_df['Qry'][0] or '.fastq.gz' in new_df['Qry'][0]:
        new_df['Ref'] = new_df['Ref'].apply(lambda x: x.split('/')[-1][:-9])
        new_df['Qry'] = new_df['Qry'].apply(lambda x: x.split('/')[-1][:-9])
    elif '.fna.gz' in new_df['Qry'][0]:
        new_df['Ref'] = new_df['Ref'].apply(lambda x: x.split('/')[-1][:-7])
        new_df['Qry'] = new_df['Qry'].apply(lambda x: x.split('/')[-1][:-7])
    elif '.fa.gz' in new_df['Qry'][0]:
        new_df['Ref'] = new_df['Ref'].apply(lambda x: x.split('/')[-1][:-6])
        new_df['Qry'] = new_df['Qry'].apply(lambda x: x.split('/')[-1][:-6])
    elif new_df['Qry'][0][-3:] == '.fa':
        new_df['Qry'] = new_df['Qry'].apply(lambda x: x.split('/')[1][:-3])
        new_df['Ref'] = new_df['Ref'].apply(lambda x: x.split('/')[1][:-3])
    elif new_df['Qry'][0][-6:] == '.fasta' or new_df['Qry'][0][-6:] == '.fastq':
        new_df['Qry'] = new_df['Qry'].apply(lambda x: x.split('/')[1][:-6])
        new_df['Ref'] = new_df['Ref'].apply(lambda x: x.split('/')[1][:-6])
    else:
        pass
    current_path = os.getcwd()
    txt_path = current_path + '/bindash_distance.txt'
    new_df.to_csv(txt_path, index=False, sep='\t')
    data = {}
    with open(txt_path, 'r') as f:
        next(f)
        for line in f:
            seq1, seq2, distance = line.strip().split()
            if seq1 not in data:
                data[seq1] = {}
            if seq2 not in data:
                data[seq2] = {}
            data[seq1][seq2] = float(distance)
            data[seq2][seq1] = float(distance)
    if os.path.exists(txt_path):
```

```
            os.remove(txt_path)
    else:
            print(f"The file {txt_path} does not exist")
    n_seqs = len(data)
    seq_names = sorted(data.keys())
    with open('bindash_dist_matrix.phy', 'w') as f:
            f.write(str(len(seq_names)) + '\n')
            for i in range(n_seqs):
                    f.write(seq_names[i])
                    for j in range(n_seqs):
                            if i == j:
                                    f.write('\t{:.5f}'.format(0.0))
                            else:
                                    if seq_names[j] not in data[seq_names[i]]:
                                            data[seq_names[i]].setdefault(seq_names[j], 0)
                                    jaccard = data[seq_names[i]][seq_names[j]]
                                    if jaccard == 0:
                                            mashD = 1
                                    else:
                                            mashD = -(1 / k) * math.log(2 * jaccard / (1 + jaccard))
                                    f.write('\t{:.5f}'.format(mashD))
                    f.write('\n')


if __name__ == '__main__':
    deal_bindash(20, 'ES29_distance.out', 'ES29_matrix.phy')
```
